# Risk of Scoliosis in Adolescent Female Rhythmic Gymnasts Compared with Healthy Peers: A Cross-Sectional Study Using the Scoliosis Tele-Screening Test

**DOI:** 10.1177/19417381261485714

**Published:** 2026-09-24

**Authors:** Ahsen Büyükaslan Bohinc, Maja Pajek

**Affiliations:** † Institute of Kinesiology, Faculty of Sport, University of Ljubljana, Ljubljana, Slovenia

**Keywords:** flexibility, generalized joint hypermobility, laxity, rhythmic gymnastics, scoliosis

## Abstract

**Background:**

Rhythmic gymnasts have a higher prevalence of scoliosis; however, the literature remains limited due to differences in methodologies, populations, and definitions. Considering the inadequacy and the inconsistency of screening methods and comparison groups in the literature, we aimed to screen rhythmic gymnasts for scoliosis risk, identify risk groups using the Scoliosis Tele-Screening Test (STS-Test), and compare them with healthy peers.

**Hypothesis:**

Rhythmic gymnasts will exhibit a different risk distribution pattern and a higher scoliosis risk score on the STS-Test compared with healthy controls.

**Study Design:**

Cross-sectional study.

**Level of Evidence:**

Level 4.

**Methods:**

Adolescents (10-18 years old) who had practised rhythmic gymnastics at a competitive level for ≥5 years (Tier 3, Participant Classification Framework) were included. A total of 56 gymnasts and 302 age-matched healthy controls were enrolled. Sport-specific data, pubertal maturity, hypermobility, STS-Test for scoliosis risk, and angle of trunk rotation were assessed.

**Results:**

Rhythmic gymnasts have higher STS-Test scores than controls (*P* < 0.001). Fisher’s exact test revealed a difference in the distribution of scoliosis risk between groups (*P* < 0.001). Post hoc analysis showed the difference was driven primarily by gymnasts having significantly more moderate-risk cases (adjusted residual [AR], 5.1; *P* < 0.001) and fewer low-risk cases (AR, –4.6; *P* < 0.001). The Healthy controls showed the opposite pattern, with significantly more low-risk cases (AR, 4.6; *P* < 0.001) and fewer moderate-risk cases (AR, –5.1; *P* < 0.001).

**Conclusion:**

Rhythmic gymnasts exhibit a higher scoliosis risk score on the STS-Test compared with healthy controls. Gymnasts had more moderate-risk and fewer low-risk cases, whereas the healthy controls exhibited the opposite pattern. Considering generalized joint hypermobility, high training volume at an early age, and risk of scoliosis, it is essential to assess scoliosis risk and closely monitor rhythmic gymnasts during growth for early diagnosis, as scoliosis can impact sports careers significantly. Gymnasts can be screened periodically for scoliosis risk using the STS-Test.

**Clinical Relevance:**

It is crucial to assess rhythmic gymnasts throughout their growth to enable early diagnosis and management of scoliosis, thereby preventing career impact.

Adolescent idiopathic scoliosis (AIS) is a 3-dimensional (3-D) complex structural deformity of the growing spine, described as a lateral deviation of the spine >10° on standing anterior-posterior radiography, accompanied by axial rotation and sagittal plane deviations.^12^ Prevalence of AIS varies between 0.35% and 5.2%; it is reported frequently as approximately 2.5% in the general population.^28^ In addition, a specific population may be more susceptible to developing AIS or to its progression, as previous studies have established a link between certain sports-specific groups and scoliosis. The association between scoliosis and rhythmic gymnastics and ballet has been known for many years, despite the limited literature reporting the presence of scoliosis in sport-specific populations.^48,64^ Scoliosis prevalence in rhythmic gymnasts varies widely, from about 12% in cohort studies,^61^ to >60% in small elite samples,^19,52^ with elevated rates compared with general population norms.^19,52,61,70^

Rhythmic gymnastics, unlike many other sports, places unique demands on the spine: it is an Olympic sport in which athletes perform choreographed routines to music while manipulating hand apparatus (rope, hoop, ball, clubs, and ribbon), combining gymnastics with elements of dance as an “aesthetic” sport.^25,54^ The sport combines high-intensity jumps, pivots, and balances with complex apparatus handling and requires extreme active and passive flexibility, high-speed turns, and precise postural control.^25^ Movements with an extreme range of motion are characteristic of rhythmic gymnastics and essential for successful performance, as higher levels of competition are associated with greater flexibility, which increases over time.^6,11^ The current “FIG Code of Points” (International Gymnastics Federation technical rules governing rhythmic gymnastics routines) encourages large-amplitude spinal movements, including repeated hyperextension and lateral flexion of the trunk, often executed on a single supporting leg.^23^ In addition, rhythmic gymnasts frequently rely on the same dominant lower limb during take-off and landing in jumps and balances, and on a dominant upper limb during apparatus throws and catches, which can lead to persistent unilateral loading patterns of the spine and pelvis.^6,22^ Furthermore, early sport specialization and high training volumes are common practices in rhythmic gymnastics, with training averaging 18 to 20 hours per week in early childhood and increasing to 40 h per week among international-level adolescent athletes.^4,25,34,69^

The occurrence of scoliosis in rhythmic gymnastics is attributed to the coincidence of 3 factors known as the “dangerous triad”: generalized joint laxity and/or generalized joint hypermobility (GJH); a heritable predisposition; delayed growth and maturity caused by physical, dietary, and psychological stresses; and persistent asymmetric spinal overloading.^48,61^ Children with GJH who have a strong ability to adapt might be drawn to gymnastics because they can meet the demands of the sport. Consequently, girls with a high GJH might have an increased vulnerability to developing AIS.^48^ However, according to the recent systematic review by Shere and Clark,^58^ there was no convincing population-based evidence for an association between hypermobility and AIS. Although populations at the extremes of hypermobility, such as dancers and rhythmic gymnasts, had a high prevalence of spinal curvature, these studies are at high risk of confounding due to variation in hypermobility measurement methods.^58^ Therefore, another possibility is that hypermobility may be one of a constellation of traits associated with AIS in these populations.^58^

Although scoliosis is more prevalent among rhythmic gymnasts, the existing literature remains limited. Neither the prevalence nor the etiopathogenesis of idiopathic or sport-associated scoliosis could be determined definitively in rhythmic gymnasts.^58,64^ Studies show inconsistent rates (15% to 40%) due to heterogeneous screening methods (Adams forward-bending test vs radiograph), populations (age and/or competitive level), definitions (Cobb ≥10° vs <10°), and regional screening practices. Therefore, scoliosis prevalence studies among gymnasts differ widely in their methods, populations, and even in definitions.^52^ The only research in the literature that compared rhythmic gymnasts with healthy controls for scoliosis using radiographic evaluation did not report the number of participants in the healthy group.^61^ Yet, it is used as a reference in this topic because it is the only radiographic rhythmic gymnast-control study (though the healthy control sample size is unreported). Despite its age, it remains a key reference, as no newer equivalent has been published and recent reviews still confirm persistent methodological gaps.^49,61^ In other studies, either the comparison group was missing, the methods were not accepted in the scientific literature or clinical practice for scoliosis screening, or the methods had poor accuracy and sensitivity.^49^

Previous screening methods were prone to higher false-positive rates.^52^ While it is neither necessary nor feasible to obtain radiographs for all rhythmic gymnasts to diagnose scoliosis, it may be more valuable to identify risk groups and implement periodic screenings using an appropriate method for those at risk. Recently, researchers developed the Scoliosis Tele-Screening Test (STS-Test) for the early detection of scoliosis risk.^66^ Unlike typical nonradiographic screening methods, the STS-Test was 94.97% accurate, 83.51% sensitive, and 98.87% specific when tested for agreement with scoliosis Cobb angle measurements on spine radiographs and with physician examination.^66^ The STS-Test evaluates risk factors (e.g., family history, sex, menarche status) and aesthetic deformities (e.g., shoulder and pelvic asymmetry, scapular prominence, and Adams forward-bending test). Items are scored to classify individual athletes into low, moderate, or high-risk categories. The STS-Test includes the major elements of the typical clinical examination of scoliosis. In addition to clinicians, it enables parents or coaches to actively participate in early detection of scoliosis by screening children periodically for scoliosis risk, as parent and physician assessments have been shown to be in agreement.^66^ Considering the inadequacy and inconsistency of screening methods and comparison groups in the literature, the STS-Test may be particularly useful for populations at risk - such as rhythmic gymnasts and ballet dancers - who are at risk of developing scoliosis.

## Aims and Objectives

This study aimed to:

- screen rhythmic gymnasts for scoliosis risk using the STS-Test and identify risk groups based on scoliosis-related risk factors;
- compare rhythmic gymnasts with healthy peers for scoliosis risk using the STS-Test; and
- use a validated assessment tool comparable with radiographic evaluation to support these objectives.

## Methods

This was a cross-sectional study. Written informed consent was obtained from the parents or legal guardians of participants <18 years old, and assent was obtained from the children themselves. Participants were only included if both consent and assent were provided. Ethics approval was obtained from the local ethics committee.

### Participants

#### Inclusion and Exclusion Criteria

Included participants were (1) adolescents aged 10 to 18 years; (2) practiced rhythmic gymnastics with ≥5 years (recreational or competitive level) experience; (3) classified as Tier 3 or higher according to the Participant Classification Framework (PCF).^47^ PCF is a 6-tiered system that classifies athletes across a spectrum of exercise backgrounds and athletic abilities. The PCF uses training volume and performance metrics to classify a participant into one of the following: Tier 0, sedentary; Tier 1, recreationally active; Tier 2, trained/developmental; Tier 3, highly trained/national level; Tier 4, elite/international level; or Tier 5, world class.^47^

Participants were excluded if they had any of the following: (1) a history of spine surgery; (2) major lower extremity trauma with associated surgery; (3) infectious, tumoral, rheumatic, or neurologic diseases affecting the spine; (4) leg length inequality >1 cm, as determined clinically using the leg length discrepancy scoliometer test.^15^ If the trunk rotation angle at the sacral base level was not 0°, further assessment was performed using a standard approach described widely in the literature^27,35,37,51^:

- direct measurement with a measuring tape (iliac spine to medial malleolus);
- indirect measurement with a measuring tape (umbilicus to medial malleolus).

A total of 56 adolescent female athletes who practice rhythmic gymnastics at the recreational or competitive level were included in the study. A total of 302 healthy peers of the same age group who were not involved in sports were included as the control group. Since rhythmic gymnastics is a discipline for girls only, the healthy control group consisted of girls as well.

### Measurement Instruments for Gymnasts

Demographic data, including name, date of birth, height, weight, age at menarche, presence of twin siblings, scoliosis, and family history, were recorded.

Sport-specific data, including years of experience, weekly training hours, and hours per session, were also recorded. Training intensities to classify the subgroups for further analysis are based on the PCF.^47^

Tanner Pubertal Maturity Stage (1-5) is a standardized system that was used for measuring and categorizing the physical development of children and adolescents during puberty, where Stage 0 denotes prepubertal status and Stages 1 to 5 reflect progressive pubertal maturation based on the development of secondary sexual characteristics.^5,45,46^ The scale comprises 6 stages ranging from no pubertal development (Stage 1) to full adult maturity (Stage 5). It focuses primarily on changes in the development of the breasts and genitals in girls and the genitals in boys, as well as other secondary sexual characteristics such as body hair growth and changes in body shape. Throughout these stages, other changes occur, such as the growth of pubic hair, changes in body fat distribution, and the growth spurt that accompanies puberty. The self-rated Tanner stage was used in this study to maintain participants’ privacy and dignity. Self-reporting of the pubertal stage still has a role in large community-based studies, as mentioned in the literature, because Tanner staging by a clinician is often not possible.^5^

GJH was assessed using the 9-point Beighton hypermobility score, with a cut-off at ≥6 points.^9,10,44^ The score consists of 5 maneuvers, 4 of which are assessed bilaterally (1 point per side), and 1 is assessed as a single item (1 point). The components of the Beighton hypermobility score are presented in Table 1.^9,10,18,44,59^

**Table 1.** Beighton hypermobility score^8,9^

GJH assessment items	Left	Right
(1) Passive dorsiflexion and hyperextension of the fifth MCP joint beyond 90 degrees	1	1
(2) Passive apposition of the thumb to the flexor aspect of the forearm	1	1
(3) Passive hyperextension of the elbow beyond 10 degrees	1	1
(4) Passive hyperextension of the knee beyond 10 degrees	1	1
(5) Active forward flexion of the trunk with the knees fully extended so that the palms of the hands rest flat on the floor	1	
Total out of 9		

GJH, generalized joint hypermobility.

Scoliosis screening was administered by a physical therapist using the STS-Test, which includes drawing-based images of body asymmetries.^66^ The STS-Test comprises 2 domains: risk factors and the existence of aesthetic deformities (Table 2). The risk factor domain includes 3 items assessing factors associated with scoliosis development, while the aesthetic deformity domain includes 7 items evaluating the presence of body asymmetries observed in scoliosis. The test incorporates drawing-based images of body asymmetries and a standard screening method - the Adams forward-bending test - to assess scoliosis-associated risk. Total scores are obtained by summing the 10 items and interpreted according to risk categories: low risk (female, 0-4 points; male, 0-3 points), moderate risk (female, 5-11 points; male, 4-8 points), and high risk (female, 12-15 points; male, 9-13 points).^66^

**Table 2.** Scoliosis Tele-Screening Test (STS-Test) items and risk classification^66^

STS-TEST	Score	Risk category	Female, points	Male, points
Risk factors		Low risk	0 to 4	0 to 3
(1) Positive family history	1			
(2) Female sex	1			
(3) No menarche, or <1 year since menarche	1			
Aesthetic deformity		Moderate risk	5 to 11	4 to 8
(4) Scapula asymmetry	1			
(5) Difference in shoulder levels from the anterior	2			
(6) Difference in shoulder levels from the posterior	1	High risk	12 to 15	9 to 13
(7) Height difference in the pelvis from the anterior	2			
(8) Height difference in the pelvis from the posterior	1			
(9) Pelvic shifting to one side from the posterior	1			
(10) Positive Adams’ test	4			

The angle of trunk rotation (ATR) was assessed with a scoliometer during the Adams forward-bending test at multiple spinal levels between T1 and S1 (cervicothoracic, thoracic, thoracolumbar, and lumbar regions), and the highest value in each region was recorded. The ATR indicates the deviation in the transverse plane caused by vertebral rotation at that segment. The scoliometer (Orthopedic Systems Inc) was used for all ATR measurements. The scoliometer is a standard tool used in scoliosis screening for >30 years, with excellent reliability (intra- and inter-rater intraclass correlation, 0.86-0.97) and acceptable validity for screening purposes (ATR-Cobb *r* = 0.46-0.54; cutoff ≥5° to 7°).^3,17^

### Statistical Analysis

Continuous data were summarized as means and standard deviations, while nonparametric data were presented as frequencies and percentages. The normality of continuous data was assessed using the Shapiro-Wilk and Kolmogorov-Smirnov tests. The Mann-Whitney *U* test was used to compare groups with respect to age, body mass index (BMI), and STS-Test scores. Fisher’s exact test was used to compare the distribution of risk categories (low, moderate, high) across groups and to assess whether the risk distribution patterns differed significantly between groups. Spearman’s rank correlation coefficient was used to determine correlations between parameters. Statistical significance was set at *P* < 0.05. Statistical analyses were performed using IBM SPSS Statistics Version 28 (IBM Corp).

## Results

Descriptive characteristics of gymnasts are shown in Table 3. The STS-Test was administered to 56 gymnasts and 302 healthy peers. Table 4 lists participant risk classification outcomes. Groups did not differ statistically in age or BMI (Table 4). Rhythmic gymnasts have higher STS-Test scores compared with age-matched healthy controls (*P* < 0.001) (Table 4).

**Table 3.** Characteristics of rhythmic gymnasts

	Rhythmic gymnast group (n = 56)
	Mean ± SD
Age at menarche	13.42 ± 1.35
Menarche, %	54.3
Dysmenorrhea, %	26.31
Tanner stage	3.03 ± 1.06
Beighton hypermobility score	6.51 ± 1.84
Age at starting gymnastics, years	5.80 ± 1.64
Gymnastics experience, years	7.83 ± 2.39
Training hours per week	14.03 ± 6.78
Training days per week	4.08 ± 0.94
Session duration, hours	3.31 ± 0.78
PCF tier, %	
Tier 3	46
Tier 4	54
Positive family history of scoliosis, %	11.4
ATR (sacrum), deg	0.54 ± 0.85
ATR (lumbar), deg	2.45 ± 2
ATR (thoracolumbar), deg	1.11 ± 1.51
ATR (thoracic), deg	2.14 ± 3.06
ATR (cervicothoracic), deg	0.4 ± 0.97

ATR, angle of trunk rotation (scoliometer degree over the segment), PCF, Participant Classification Framework.

**Table 4.** Scoliosis risk distribution and comparison of scoliosis risk between groups

	Rhythmic gymnast group (n = 56)	Healthy control group (n = 302)	*P* value (*U*)
Age, years	13.63 ± 2.25	13.10 ± 2.23	0.19
BMI, kg/cm^2^	18.55 ± 2.17	19.3 ± 3.8	0.04*
STS-Test score	5.34 ± 2.70	3.41 ± 3.39	0.001*
STS-Test risk groups			
Low risk, n (%)	19 (34.5)	226 (74.8)	
Moderate risk, n (%)	35 (62.1)	60 (19.9)	
High risk, n (%)	2 (3.4)	16 (5.3)	

Data values are mean ± SD unless otherwise stated. BMI, body mass index; STS-Test, scoliosis tele-screening test, *U*, Mann-Whitney *U* test.

* Statistically significant at *P* ≤ 0.05.

**Figure 1.**
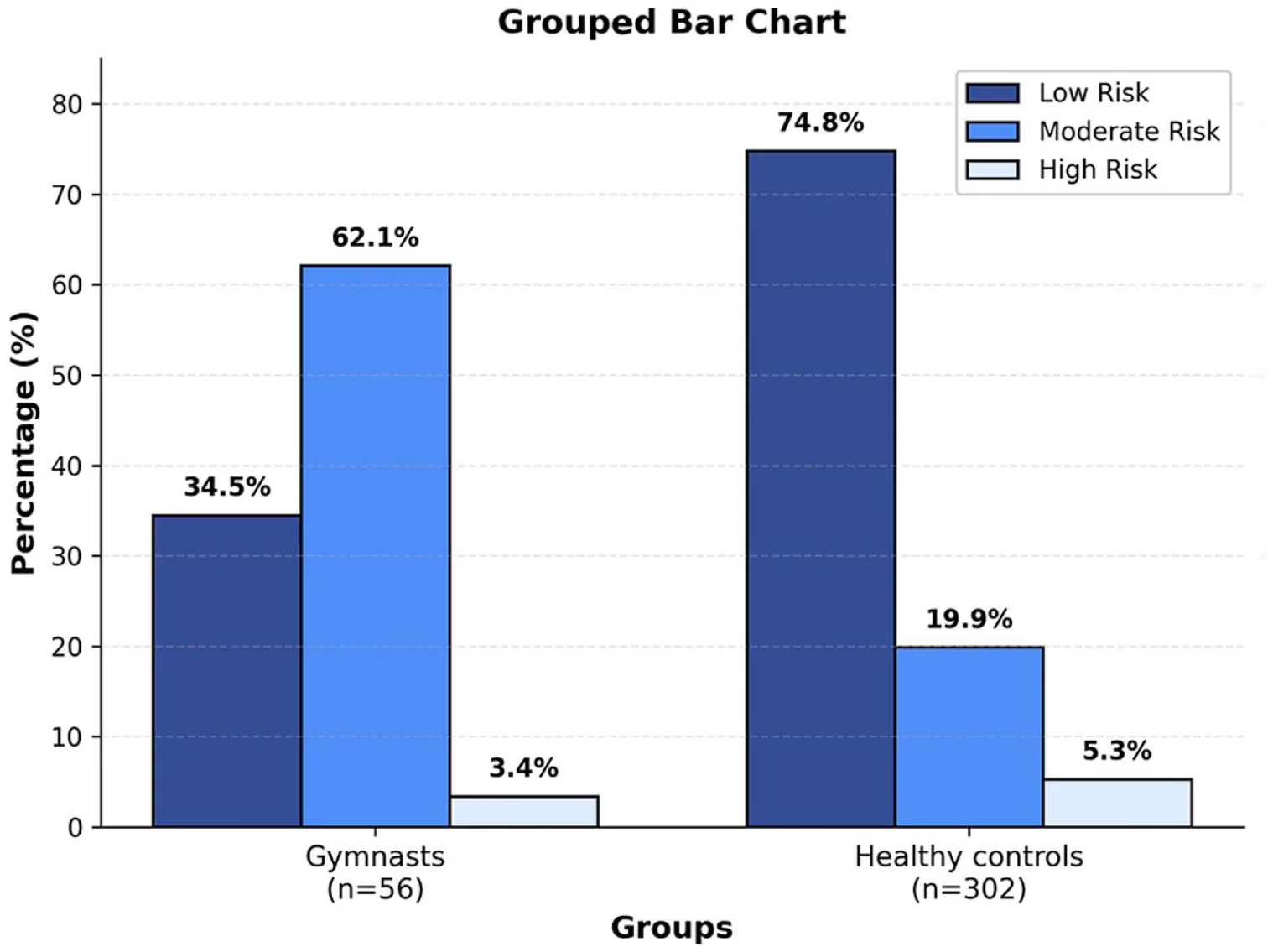
Scoliosis risk distribution comparison between groups (Fishers exact test, *P* < 0.001). Key findings: gymnasts, 62.1% (moderate risk); healthy controls, 74.8% (low risk; *P* < 0.001 (highly significant).

Fisher’s exact test revealed a significant difference in the distribution of scoliosis risk between the 2 groups (*P* < 0.001) (Figure 1). Given the significant difference, a post hoc analysis was conducted to identify which specific categories drove the difference. Post hoc analysis using adjusted standardized residuals revealed that the significant difference was driven primarily by the following factors: the gymnasts group had significantly more moderate-risk cases than expected (adjusted residual [AR], 5.1; *P* < 0.001) and fewer low-risk cases than expected (AR, –4.6; *P* < 0.001). The healthy control group showed the opposite pattern, with significantly more low-risk cases (AR, 4.6; *P* < 0.001) and fewer moderate-risk cases than expected (AR, –5.1; *P* < 0.001). No significant differences were observed in the high-risk category between the 2 groups (*P* ≥ 0.05).

When the gymnasts were divided into 2 groups based on PCF tier, a statistically significant difference was observed between Tiers 3 and 4 in the STS-Test (*P* = 0.02). In addition, the STS-Test score was correlated with weekly training hours; as weekly training hours increased, the STS-Test risk score increased (*P* = 0.01, *r* = 0.456). STS-Test score was also correlated with ATR (lumbar) and ATR (thoracic) (*P* = 0.008, *r* = 0.481; *P* = 0.001, *r* = 0.585, respectively).

## Discussion

The principal finding of this study was that rhythmic gymnasts had a higher scoliosis risk score on the STS-Test compared with healthy controls. Moreover, the gymnasts group had significantly more moderate-risk cases and fewer low-risk cases, whereas the healthy control group showed the opposite pattern, with substantially more low-risk cases and fewer moderate-risk cases.

Multiple factors, including greater height, delayed puberty, and late menarche in girls, low BMI, abnormal leptin bioavailability, and GJH, have been established to contribute to the initiation or progression of AIS.^7,12,16,58^ Among the structural and biomechanical factors, spinal sagittal alignment has been proposed as a key contributor.^33,40,42,43,50,57^ According to biomechanical theory, the hypokyphotic spine initiates scoliosis development by enhancing rotation under dorsal shear loads in backwards-inclined segments, reducing facet joint stability.^21,31,63^ In rhythmic gymnasts, the characteristic flat back and hypokyphotic spine may be particularly relevant. Rhythmic gymnasts typically present with a flat back posture and a lack of physiologic sagittal curvature of the spine, and typical movements with excessive hyperextension of the thoracic spine may be initiators or contributors to scoliosis development in these athletes.^60^ Burwell and colleagues^13,19^ emphasized that delayed puberty in rhythmic gymnasts and ballet dancers does not necessarily invalidate the neuro‑osseous timing of maturation hypothesis for idiopathic scoliosis because scoliotic changes in these athletes might represent a distinct sport‑associated scoliosis driven by environmental and/or exercise patterns rather than typical idiopathic scoliosis, proposing that multiple etiologic pathways may coexist.^13,19^ However, the existence of multiple etiologic pathways and their interactions and influences on scoliosis development or progression remains unclear.^12-14,21^

In this study, we found that, as weekly training hours increased, the scoliosis risk score in the STS-Test increased. Moreover, when the gymnasts were divided into 2 groups by PCF tier, Tier 4 had a higher STS-Test score than Tier 3, consistent with the association between training volume and scoliosis risk. This is similar to the study by Watanabe et al,^64^ which found that the odds of AIS increased with training frequency, years of experience, and training duration in ballet.^64^ Both ballet and rhythmic gymnastics are associated with GJH and excessive spinal hyperextension in routines. The increased risk of scoliosis among rhythmic gymnasts may be related to GJH, which allows an extreme range of motion, particularly excessive hyperextension of the spine. While extreme hyperextension of the spine has an aesthetic and performance advantage in rhythmic gymnastics, it may contribute to hypokyphotic deformity and derotation, thereby progressing 3-D deformity. Nevertheless, it remains unclear why rhythmic gymnasts have a higher incidence of scoliosis; perhaps the extreme hypokyphotic nature of repeated movement contributes to progression and leads to the considerable severity of the deformity at a detectable level.^64^

Research on the prevalence of scoliosis among rhythmic gymnasts varies due to differences in methods, populations, and definitions. Detection methods consist of radiologic diagnosis in some studies, clinical screening with the Adams forward bending test or scoliometer in others, and topography tools or Spinal Mouse for postural metrics in others, with no typical comparison with controls.^13,19,26,52,56,61^ Screening tools such as ATR on scoliometer (≥5° or >7° cutoffs) yield higher false-positive rates because many cases lack radiographic confirmation, whereas studies that require radiographic confirmation after screening (Adams forward bending test to radiograph) report lower, more comparable scoliosis prevalence rates.^2,32,36,60,62,67^ Tanchev et al^61^ reported a 10-fold higher incidence (12%) of scoliosis among rhythmic gymnasts compared with their age-matched controls (1.1%).^61^ Tanchev’s study is the only study that used the gold-standard radiographic diagnostic method to compare rhythmic gymnasts with healthy peers and assess the presence of scoliosis.^61^ It compared 100 rhythmic gymnasts with “normal girls not involved in sports”; it lacks an adequate control sample size, age details, recruitment methods, and detailed baseline metrics. Therefore, several specific control-group details are reported inadequately.^61^ In contrast, the present study did not diagnose scoliosis; instead, it screened for scoliosis risk using the STS-Test.^66^ The present study used a new method, the STS-Test, which combines aesthetic evaluation with the Adams forward-bending test, sex, menarche, and family history of scoliosis, thereby increasing screening accuracy.^66^ The STS-Test has been shown to be 94.97% accurate, 83.51% sensitive, and 98.87% specific for detecting scoliosis, comparable with the clinician’s examination in a previous study.^66^ While the positive predictive value (PPV) for the STS-Test alone was 96.20%, the highest PPV was 81.0% when combining the Adams forward bending test, scoliometer measurement, and Moiré topography.^24,29,30,39,41,53,65,68^

STS-Test is valuable for identifying risk groups and may also be useful for periodic check-ups for those at risk. During periods of rapid growth, rhythmic gymnasts’ individual risk of scoliosis should be monitored closely, with clinicians, parents, and coaches evaluating risk every 6 months using the STS-Test. This approach enables timely identification of at-risk athletes and supports early preventive measures. Although school-based scoliosis screenings continue in some countries today, their effectiveness, clinical utility, and cost-effectiveness remain debated.^1,20,38,55^ Considering that rhythmic gymnasts constitute a high-risk group, such screenings should be targeted specifically at athletes in this population.

### Limitations

The cross-sectional design of this study limits the ability to infer causal relationships between training volume, GJH, and scoliosis risk. In addition, the inclusion of only female rhythmic gymnasts with ≥5 years of experience and at Tier 3 or higher restricts any generalizing of the findings to male athletes, athletes in other sports, or those practicing at recreational levels. Although the statistical analyses accounted for the unequal group sizes, the difference in sample numbers between gymnasts and controls should still be considered when interpreting the generalizability of the results.

## Conclusion

Rhythmic gymnasts exhibit a higher scoliosis risk score on the STS-Test compared with age-matched healthy controls. In terms of distribution, rhythmic gymnasts had more moderate-risk and fewer low-risk cases, whereas the healthy control group exhibited the opposite pattern, with more low-risk and fewer moderate-risk cases. In addition, higher weekly training hours were associated with increased STS-Test scores. Considering GJH, high training volume at an early age, and the elevated risk of scoliosis in this population, regular assessment and close monitoring during the growth period are essential for early diagnosis and management of scoliosis progression and deformity-related complications. The STS-Test can serve as a practical screening tool, and clinicians, parents, and coaches can contribute to early detection through periodic assessments. Future research should focus on repeated testing of at-risk athletes to optimize preventive strategies.
